# Development of squaraine based G-quadruplex ligands using click chemistry

**DOI:** 10.1038/s41598-017-04344-x

**Published:** 2017-07-06

**Authors:** Xin Zhang, Yongbiao Wei, Tao Bing, Xiangjun Liu, Nan Zhang, Junyan Wang, Junqing He, Bing Jin, Dihua Shangguan

**Affiliations:** 1 Beijing National Laboratory for Molecular Sciences, Key Laboratory of Analytical Chemistry for Living Biosystems, Institute of Chemistry, Research/Education Center for Excellence in Molecular Sciences, Institute of Chemistry, Chinese Academy of Sciences, Beijing, 100190 China; 2 University of the Chinese Academy of Sciences, Beijing, 100049 China

## Abstract

The G-quadruplex (G4) structures of nucleic acids are considered to play an intrinsic role in gene expression. To this end, the development of new G4 ligands has attracted extensive research interests towards potential applications as G4-targeted drugs and molecular probes. To date, the majority of G4 ligands have been composed of an extended planar aromatic scaffold that interacts with the terminal G-tetrad plane via *π-π* interactions, and various side chains that interact with the sugar-phosphate backbone, loops or grooves of the G4 structures. The side chains act to modulate the affinity and selectivity of the G4 ligands, alongside influencing their biodistribution. Here, we present a click chemistry methodology to generate a series of squaraine-based G4 ligand derivatives based on our previously reported G4 probe (named CSTS) but with varing side chains. We find that importantly these new G4 ligand derivatives retain the G4 selectivity, optical properties and low cytotoxicity of CSTS, but exhibit different binding behaviors to G4 structures, and distinct cellular uptake efficiencies. Indeed, of these new complexes, several exhibit much higher affinity and cellular uptake than CSTS. Overall, this novel, facile and highly effective strategy has significant future potential for the high-throughput screening of G4 ligands or probes targeted towards *in vivo* applications.

## Introduction

Guanine-rich nucleic acids (DNA or RNA) are known to self-assemble into four-stranded structures defined as G-quadruplexes (G4s), which are composed of the planar arrangement of four guanine bases stabilized by Hoogsteen hydrogen bonds (known as a G-quartet)^1–3^. Such G4 forming sequences have been reported to be prevalent in genomes, such as immunoglobulin switch regions and promoter regions of oncogenes and telomere ends^4, 5^, as well as in particular RNA domains, such as the first introns, 5′- and 3′- untranslated regions, telomeric RNA^6^, and the genomic RNA of Zika virus replication^7^. Accumulating evidence has suggested that G4s play an intrinsic role in gene expression^8–14^.

**Figure 1:**
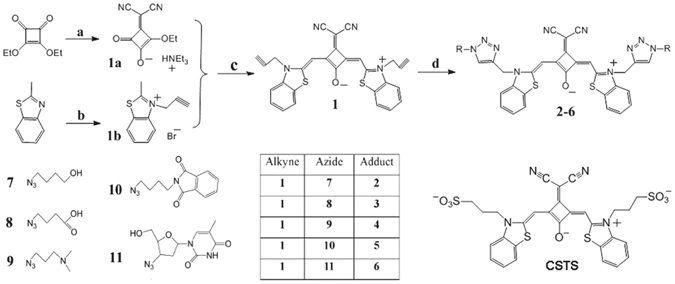
Synthetic route of compound **1–6**. Reagents and conditions: (**a**) malonodinitrile, triethylamine, benzene, room temperature, 30 min; (**b**) propargyl bromide, CH_3_CN, reflux, 8 h; (**c**) toluene, n-butanol, pyridine, reflux, 8 h; (**d**) sodium ascorbate, CuSO_4_·5H_2_O, TBTA, DMSO/H_2_O (10:1), room temperature, 3 h.

Due to their potentially significant role in biological systems, G4s are considered as promising targets for therapeutic intervention applications. Therefore, in recent years, the development of G4 ligands as chemotherapy agents or detection probes has attracted a substantial amount of effort^3, 15–22^. Arising from these studies are a wide variety of G4 ligand variants, such as acridine derivatives, bisquinolinium derivatives, cationic porphyrins, ethidium derivatives, triarylimidazole derivatives and transition metal complexes^2, 23–34^. Most commonly, the overall chemical construct of G4 ligands is comprised of an extended planar aromatic scaffold, which interacts primarily via *π*–*π* stacking to the terminal G-tetrad, alongside side chains tethered to the aromatic scaffold which interact with the sugar-phosphate backbone, loops or grooves of the G4s. Our previous report focused on a G4 ligand with fluorescent probe capacity, based on a dicyanomethylene-functionalized benzothiazole squaraine, namely CSTS (Fig. 1). Encouragingly, CSTS showed long ex/em wavelengths (680/710 nm), low background fluorescence, high fluorescence quantum yield and an excellent selectivity to parallel G4s^33^. However, the further application of CSTS in cell analysis is not possible owing to its poor cellular uptake, likely due to the two negatively charged sulfonic acid groups on the side chains impeding cell membrane penetration. The anionic character of CSTS also decreased its affinity to negatively charged G4s.

To this end, it is clear that systematically changing the side chains is a facile pathway towards increasing binding selectivity and affinity to specific G4s, as well as improving cellular uptake and localization, in particular, as the planar aromatic scaffold of CSTS determines its excellent optical characteristics and high selectivity to parallel G4s. For such an approach, it is necessary to develop a new modification strategy to readily produce a catalogue of CSTS analogues with different side chains, in particular, in order to screen new probes for G4 recognition and for *in vivo* applications. Here, we report a new, highly efficient, molecular platform based on a dicyanomethylene-substituted benzothiazole squaraine functionalized with two alkyne groups (compound **1**), which is readily coupled to various azides through a 1,3-dipolar cycloaddition reaction (click chemistry) using copper(I) as a catalyst^35, 36^. Exploiting this platform, we have synthesized five new CSTS analogues (Fig. 1) with a variety of side chains (Compounds **2**–**6**) and tested their binding behaviors to different G4s and their relative cellular interaction capacity.

## Results

### Design and synthesis

The click reaction approach, involving an azide and alkyne to yield a covalent product 1,4-disubstituted 1,2,3-triazole, has been widely used in conjugation chemistry as it provides good selectivity, applicability in aqueous and aerobic systems, tolerance to a variety of functional groups and quantitative yields^37^. In order to efficiently obtain CSTS analogues with different side-chains, we functionalized the planar aromatic scaffold of CSTS with two alkyne groups (compound **1**, Fig. 1) and used **1** as a molecular platform to couple different azides. Azides **7**–**11** which comprise positively and negatively side chains, aromatic substituents and a neutral thymine, were used to encompass several types of interaction modes, including *π*–*π* stacking, electrostatic and hydrogen bonding, with the aim of maximizing the probability of identifying a suitable G4 ligand^38^. We note that zidovudine (**11**) is a clinical drug approved by the Food and Drug Administration (FDA)^39^, it is a deoxythymidine derivative which may interact with other nucleic acid bases on the loop of G4s through hydrogen-bond interactions. Figure 1 illustrates the synthetic route for the strategically designed compounds (**1–6**).

### Absorption and fluorescence spectra

**Figure 2:**
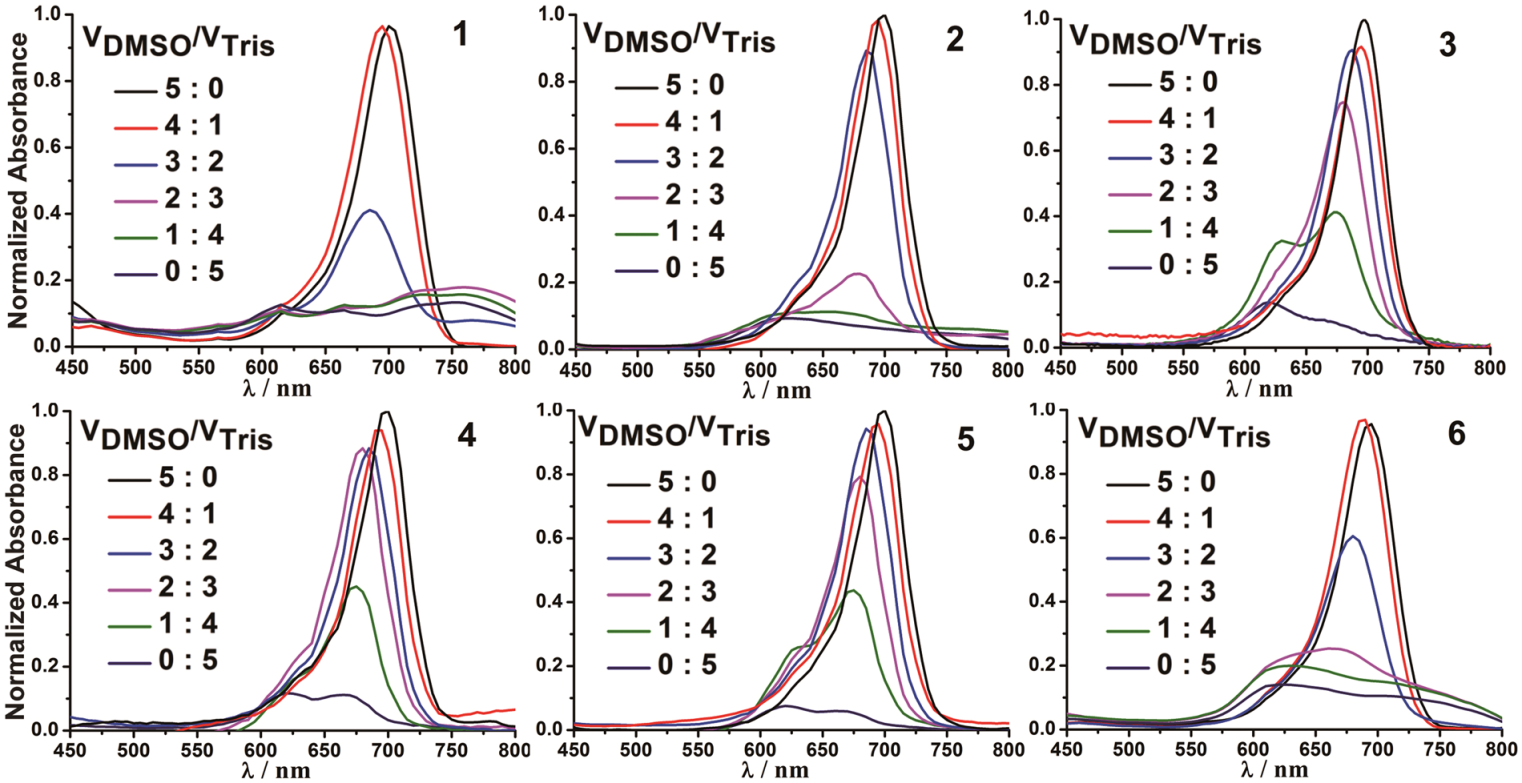
Absorption spectra of 4 μM compounds in mixed solvents of DMSO and Tris-HCl buffer.

Dye molecules with large *π*–conjugate aromatic systems are prone to self-association in aqueous media owing to intermolecular van der Waals forces and *π*–*π* stacking interactions. In order to study the aggregation behavior of compounds **1–6** in aqueous solution, the absorption and fluorescence spectra were recorded in a mixed solvent system comprised of DMSO and Tris-HCl buffer. As shown in Fig. 2, compounds **1–6** exhibit an intense absorption band with a maximum at 700 nm in DMSO, which indicates that compounds **1–6** in DMSO mainly exist in monomer form. With the increase in buffer content, the absorption band at 700 nm shows a subtle blue-shift and significantly decreases in intensity, and alongside this a new broad band at 620 nm emerges. The fluorescence spectra (Figure S1) of compounds **1–6** revealed virtually no fluorescence signal in buffer; and alongside this the fluorescence band (698–725 nm) is significantly enhanced and accompanied by a red-shift with increasing DMSO content. The absorption and emission spectral changes in the mixed DMSO and Tris-HCl buffer solvent system suggests that compounds **1–6** have a low solubility and a tendency to self-aggregate in aqueous solution. Overall, the absorption and emission spectra of **1**–**6** are similar to that of CSTS, which suggests that the alkyne group in **1** and the formed triazole moiety in **2**–**6** do not significantly affect optical characteristics. Notably, the absorption and emission spectra of these compounds in low DMSO concentration solution are vastly different, (e.g. **3**, **4** and **5** showed much stronger absorption and emission bands than the other compounds in the 20% (v/v) DMSO solution), suggesting the different water-solubility and aggregation tendency of these compounds results from the side chain characters.

### Absorption spectra in the presence of DNA

In order to investigate the selectivity of compounds **1–6** towards various DNA sequences, the absorption spectra were recorded in the presence and absence of DNA, respectively. The DNA sequences used include: a single-stranded DNA (ssa); a duplex DNA (ssab) formed by ssa and its complementary sequence; and eight different G4 sequences (see Table 1).

**Table 1:** Oligonucleotides used in this study.

Name	Sequence (from 5′ to 3′)	DNA/RNA structure
**ckit2** ^44^	CGGGCGGGCGCGAGGGAGGGT	parallel G4 DNA
**EAD** ^45^	CTGGGTGGGTGGGTGGGA	parallel G4 DNA
**Pu22** ^44^	TGAGGGTGGGTAGGGTGGGTAA	parallel G4 DNA
**Pu27** ^44^	TGGGGAGGGTGGGGAGGGTGGGGAAGG	parallel G4 DNA
**Tel22** ^46^	AGGGUUAGGGUUAGGGUUAGGG	parallel G4 RNA
**TRF2** ^47^	CGGGAGGGCGGGGAGGGC	parallel G4 RNA
**NS5A** ^7^	GUGGAGGUGGGACGGGAG	parallel G4 RNA
**NS5B** ^7^	UCGGAUGUGGCAGAGGGGGCUGGAG	parallel G4 RNA
**22AG in K** ^**+**^^48^	AGGGTTAGGGTTAGGGTTAGGG	mixed type/hybrid G4 DNA
**22AG in Na** ^**+**^	AGGGTTAGGGTTAGGGTTAGGG	antiparallel G4 DNA
**TBA** ^49^	GGTTGGTGTGGTTGG	antiparallel G4 DNA
**SPB1** ^50^	GGCGAGGAGGGGCGTGGCCGGC	antiparallel G4 DNA
**Tel22-mut**	AGUGUUAGUGUUAGUGUUAGUG	single stranded RNA
**ssa**	CCAGTTCGTAGTAACCC	single stranded DNA
**ssab**	ssa + GGGTTACTACGAACTGG	double stranded DNA

**Figure 3:**
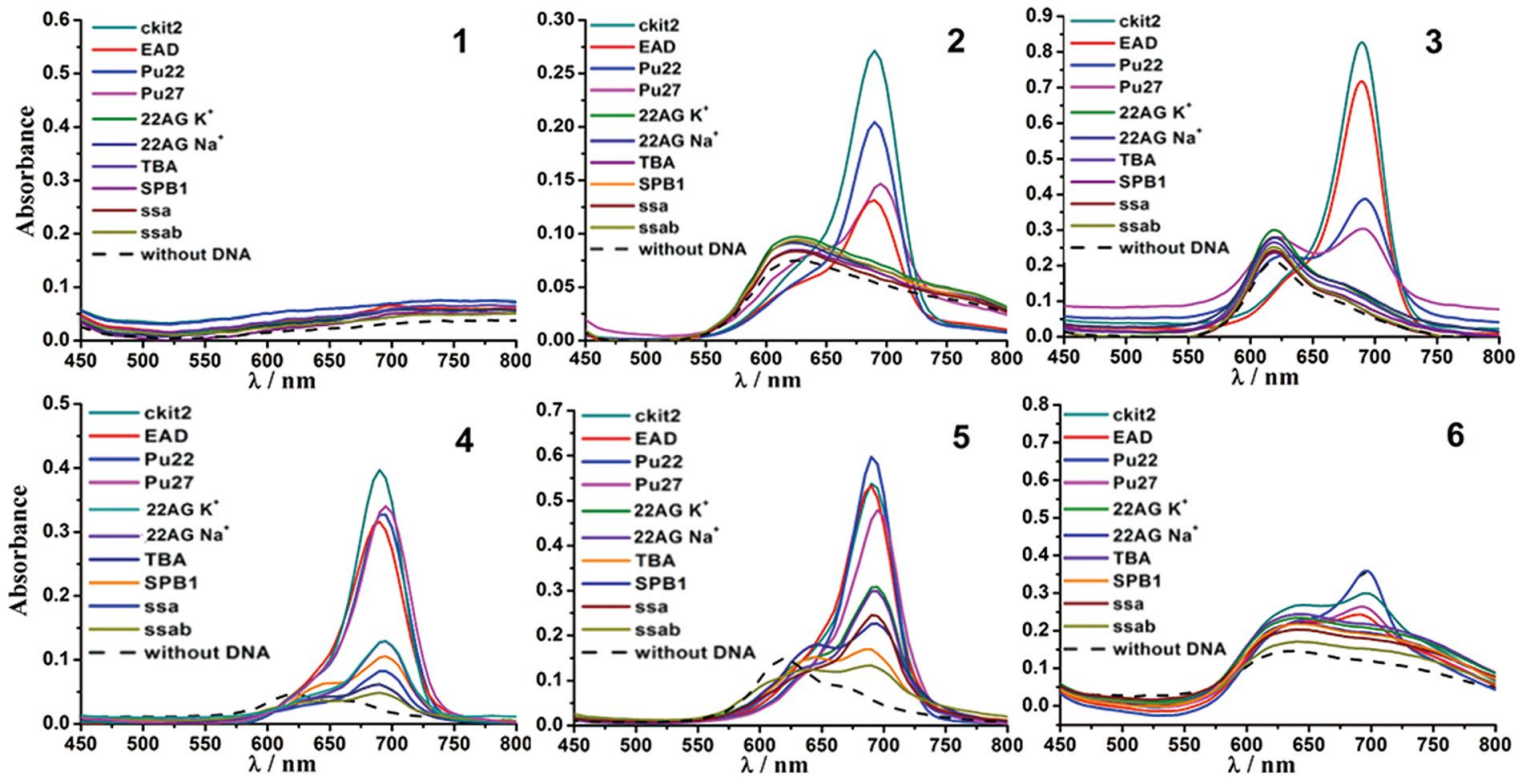
Absorption spectra of **1**–**6** (10 μM) with different DNA sequences (30 μM) in Tris-HCl buffer.

As shown in Fig. 3, in the absence of DNA, the absorption spectra of **1**–**6** in Tris-HCl buffer (dash line) display a broad absorption band (580–800 nm) (**1,2,6**) or a broad band with a peak around 620 nm and a shoulder around 680 nm (**3**, **4, 5**), which belongs to the absorption of H-aggregation and monomer forms. As anticipated, with the addition of parallel G4s (Pu27, Pu22, ckit2, EAD), the 690 nm absorption band (monomer form) of **2–6 is** greatly enhanced and accompanied by a decrease in the 620 nm absorption band intensity. The other DNA sequences, including antiparallel G4s, mixed type G4, and ssa, ssab DNA did not cause noticeable spectral changes, with the exception that **4** and **5** showed a similar but much weaker spectral change than that caused by the parallel G4s. Overall, These results suggest that the side chains in **2**–**6** (including the triazole moiety) do not change the selectivity to parallel G4s (i.e., compounds **2–6** mainly interacted with parallel G4s via *π-π* stacking). Furthermore, the weak enhancement of the 690 nm absorption band of **4** and **5** in the presence of other DNA can be attributed to slight nonspecific interactions between the side chains of **4**/**5** and DNA. For example, the amino-groups of **4** possess positive charges at physiological pH, which may electrostatically interact with the negatively charged nucleic acids backbone. Notably, the absorption spectrum of **1** was not notably changed by all of tested DNA sequences, which may due to its low solubility and high aggregation propensity in buffer.

### Fluorescence spectra in the presence of DNA/RNA

**Figure 4:**
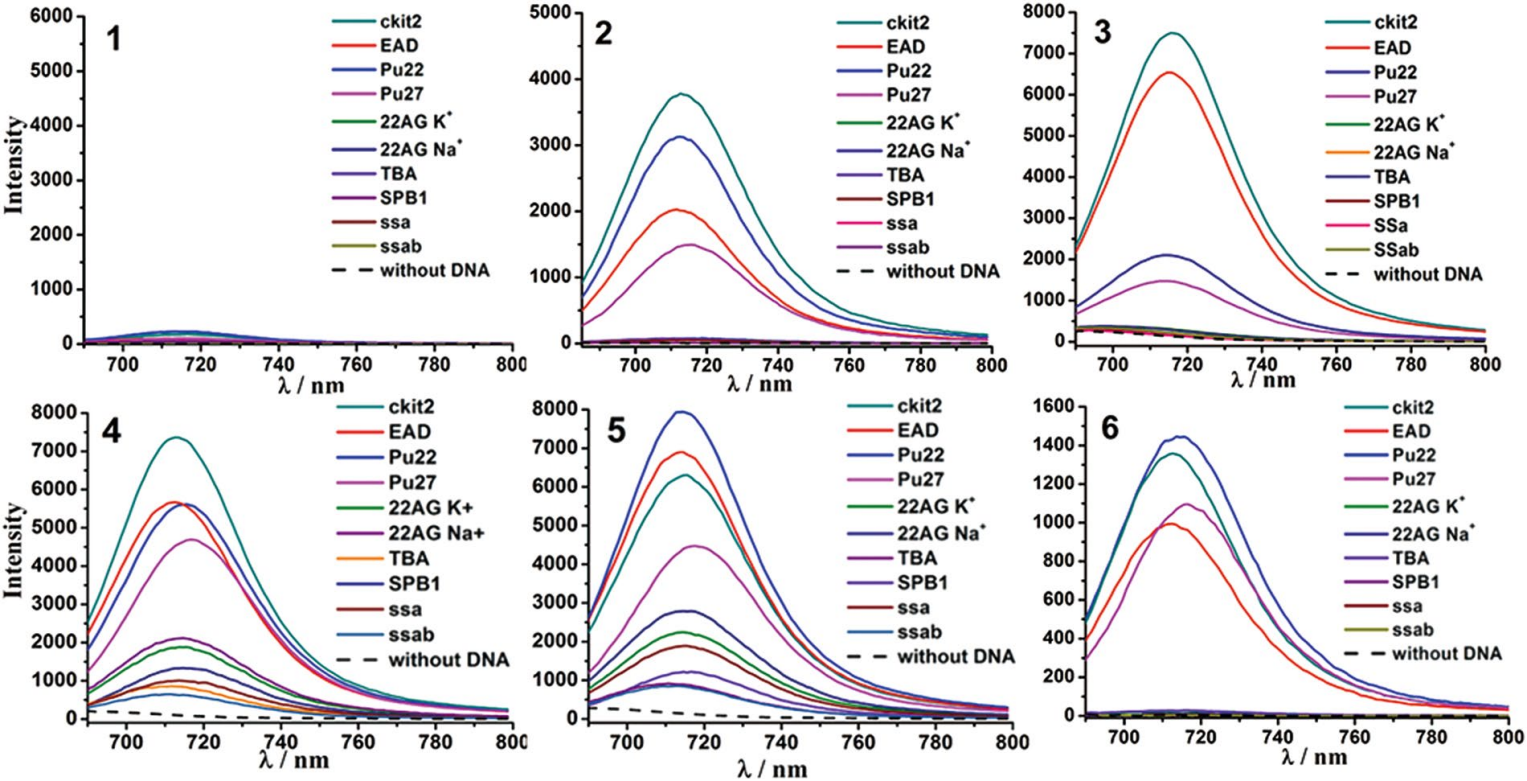
Fluorescence spectra of **1–6** (5 μM) with different DNA sequences (15 μM) in Tris-HCl buffer.

To further evaluate the interaction of compounds **1–6** with different types of DNA, their fluorescence spectra in the presence of DNA were measured with excitation at 670 nm. Overall, the fluorescence spectra changes of all of the compounds show similar tendency with their absorption changes upon the addition of DNA (Fig. 4). Notably, the free compounds do not show fluorescence in neat buffer solution. Interestingly, the addition of parallel G4s significantly enhanced the fluorescence of **2–6**. In contrast, the non-parallel G4s and non-G4 DNA did not cause significant fluorescence of these compounds, except in the case of **4** and **5** displaying a similar but much weaker fluorescent enhancement than that caused by the parallel G4s. The fluorescence spectra of the compounds in the presence of different RNA were also measured (Figure S2). Strong fluorescence was observed in the presence of parallel G4s (Tel, TRF, NS5A, NS5B) and very weak or no fluorescence was observed in the presence of non G4 RNA (Tel-mut). These results, in particular the varying extent of spectral enhancement caused by different G4s, suggest diverse affinities to the tested G4s.

Since ckit2 or Pu22 caused a strong enhancement of absorption and fluorescence spectra in compound **2–6**, the fluorescence response of these compounds to ckit2 or Pu22 were further investigated by fluorimetric titrations. Overall, we find that the fluorescence signal is gradually enhanced upon the addition of ckit2 or Pu22 (Figure S3). The titration curves were fitted to an independent-site model (eqn (1), Experimental)^40^ and the resultant apparent binding constants (*K*_a_) and stoichiometries are shown in Table 2. The titration data of **2**, **3** and **6** fit well using a 1:1 binding mode (compounds:G4) and the titration data of **4** and **5** using a 2:1 binding mode (compounds:G4). As compounds **4** and **5** showed a much higher affinity than the other compounds, this suggests a possible interaction of the side chains with the G4 loop. The fluorescence quantum yields (*Φ*) of compounds **2–6** (1 μM) in the presence of ckit2 or Pu22 (5 μM) were found to be in the range of 0.35–0.37 (Table 2), except compound **6** with a *Φ* value of 0.066.

**Table 2:** Binding stoichiometry [putative number of binding sites on ckit2 (*n*)], apparent binding equilibrium constants (*K*_a_), and fluorescence quantum yields (*Φ*).

Sample	n	*K*_a_ (M^−1^)	*Φ*
**2** (ckit2)	1	4.78 × 10^4^ ± 0.91	0.345
**3** (ckit2)	1	2.46 × 10^4^ ± 1.18	0.370
**4** (ckit2)	2	2.27 × 10^7^ ± 0.61	0.358
**5** (Pu22)	2	2.19 × 10^8^ ± 0.01	0.359
**6** (Pu22)	1	1.81 × 10^5^ ± 0.26	0.066

### The effect of compounds on the conformation and stability of G4s

**Figure 5:**
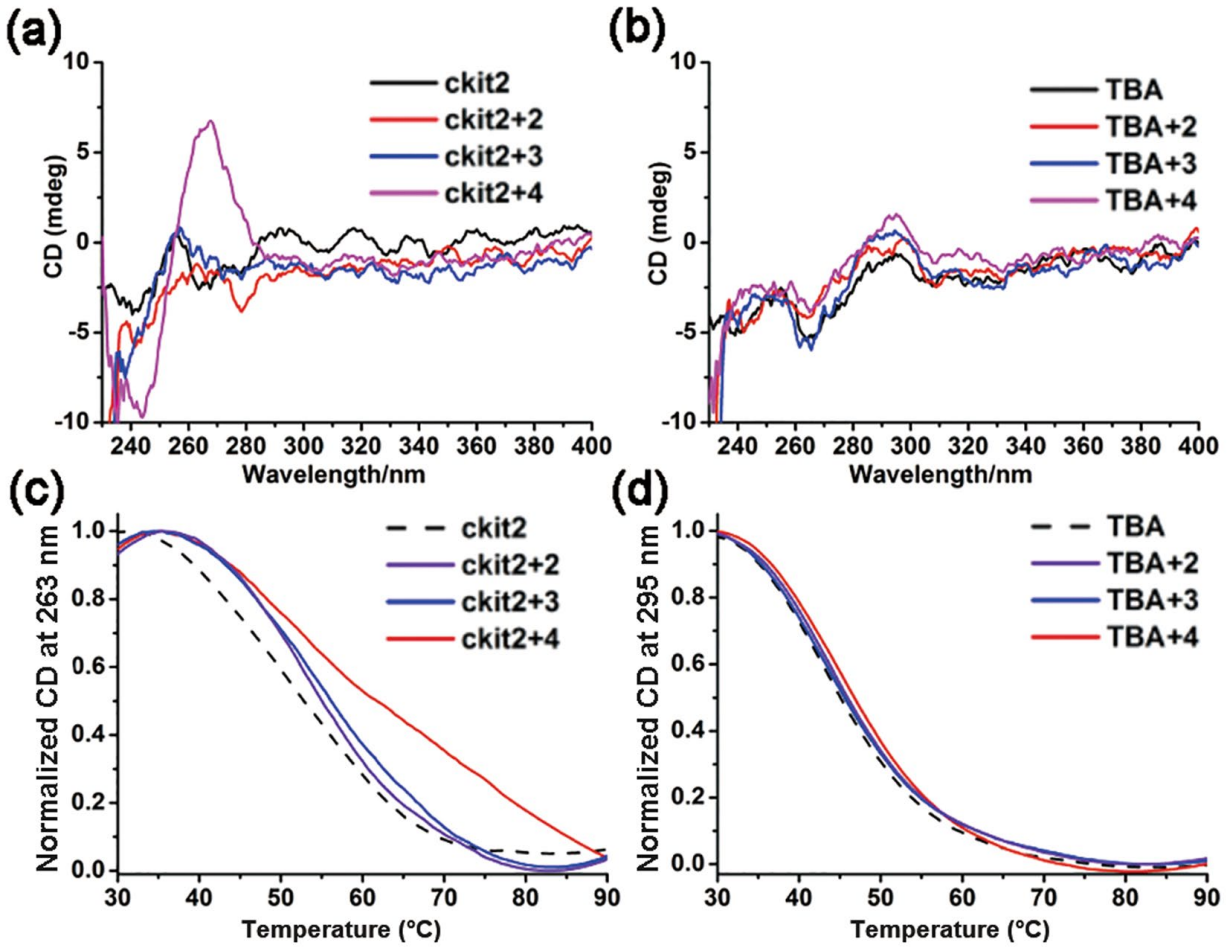
CD spectra of ckit2 (**a**) and TBA (**b**) (4 μM) in the absence and presence of compounds **2–4**(20 μM); CD melting curves of ckit2 (**c**) and TBA (**d**) (4 μM) in the presence and absence of compounds **2–4** (20 μM).

Circular dichroism (CD) spectroscopy is widely used for tracing conformational transitions of G4^41^. By this method, G4s with parallel orientation exhibit a positive CD signal around 265 nm and a negative signal around 240 nm, whereas antiparallel orientations exhibit a positive band around 295 nm and a negative band around 265 nm^42^. Overall, we find that all of the compounds do not exhibit significant CD signals due to their symmetrical structures. Compounds **2–4** were chosen to further investigate the effect on the conformation of G4s by measuring their CD spectra in the absence and presence of the ligand (Fig. 5(a,b)). The cations Na^+^ and K^+^ are known to greatly affect the G4 structure formation, therefore these experiments were performed in Tris-HCl buffer without monovalent cations (Na^+^ or K^+^). In the absence of these compounds, ckit2 and TBA did not show CD signals of the G4 structure. The addition of compounds **2** and **3** did not significantly change the CD signals of both sequences, suggesting that compounds **2** and **3** cannot induce G4 formation. However, with the addition of compound **4**, ckit2 showed distinct CD signals of parallel G4 (positive at 264 nm and negative at 245 nm), but TBA did not show notable CD change, suggesting that compound **4** can induce ckit2 to form the parallel G4 structure.

It is known that small molecules that bind to G4s may increase the stability of G4. Conveniently, the extent of G4 stabilization by the compounds can be evaluated by the extent of enhancement of G4 melting temperature^43^. Therefore, to further study the effect of the compounds on the thermal stability of G4s, we performed CD melting experiments in Tris-HCl buffer containing 5 mM K^+^. The melting curves were recorded through the change in CD signal at 263 nm for ckit2 and 295 nm for TBA. As shown in Fig. 5(c,d), the addition of compounds **2–4** did not significantly affect the melting temperature (*T*_m_) of TBA, but increased the *T*_m_ value of ckit2 by 2.0–9.0 °C (Table 3), suggesting that compounds **2–4** bind to parallel G4 ckit2 resulting in improved thermal stability. Among these compounds, compound **4** led to the highest *T*_m_ value of ckit2, suggesting a strong interaction with ckit2. Overall, these of melting experiment results are consistent with that attained in the spectra studies.

**Table 3:** Thermal stability of ckit2 and TBA with compounds **2**–**4** measured by CD melting experiment.

Compound	*T*_m_(ckit2) [°C]	*ΔT*_m_(ckit2) [°C]	*T*_m_(TBA) [°C]	*Δ T*_m_(TBA) [°C]
—	52.8	—	45.1	—
**2**	55.1	2.3	45.6	0.5
**3**	56.2	3.4	46.0	0.9
**4**	61.8	9	46.8	1.7

### Cellular interaction study

**Figure 6:**
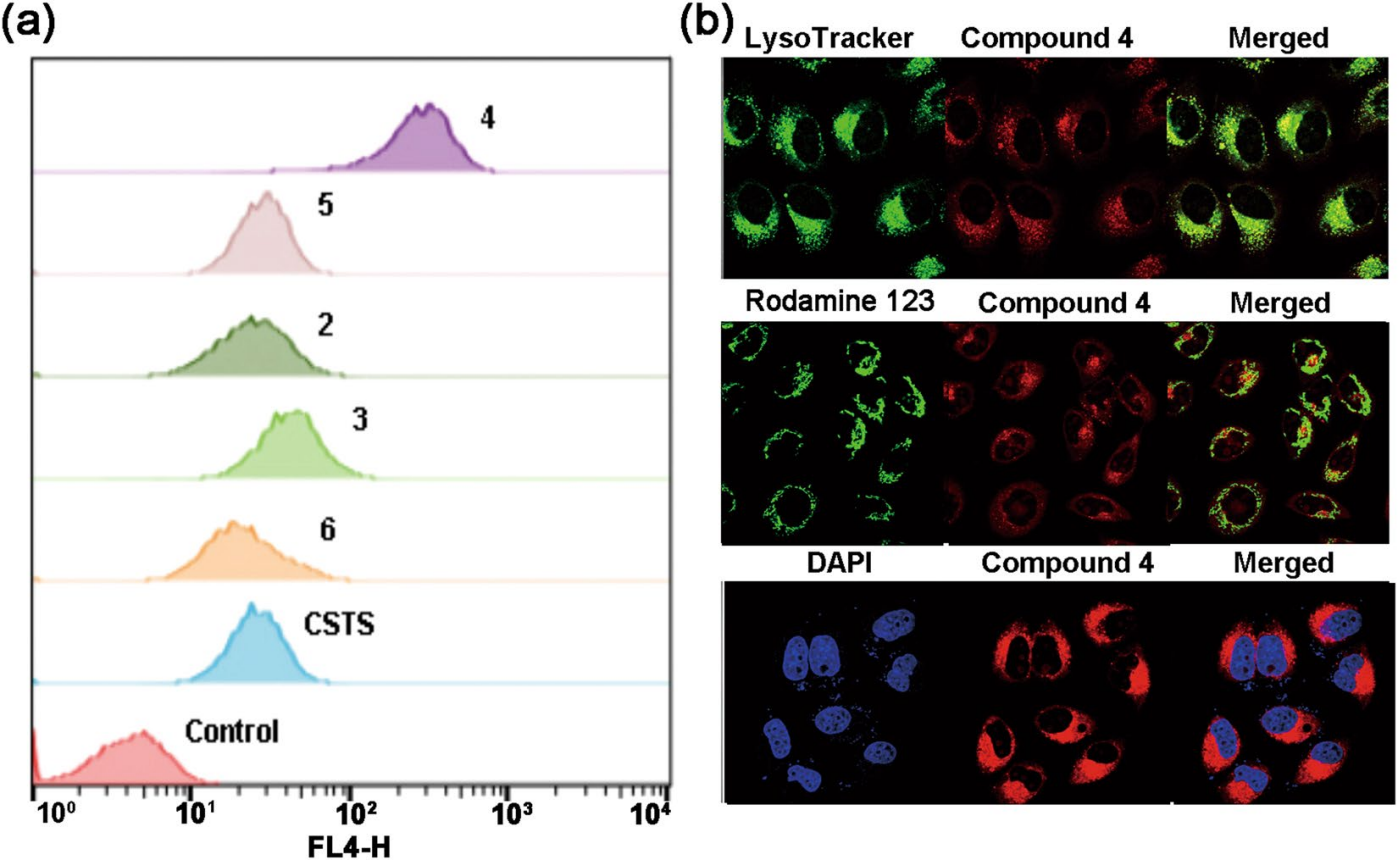
(**a**) Flow cytometry histogram of MCF-7 cells after incubated with compounds; (**b**) Confocal imaging of MCF-7 cells stained with compound **4** (2 μM) (λex = 635 nm) and Rodamine 123 (1 μM) (λex = 488 nm); compound **4** (2 μM) (λex = 635 nm) and Lyso Tracker Green DND-26 (10 μM) (λex = 488 nm); compound **4** (4 μM) (λex = 635 nm) and DAPI (10 μM) (λex = 405 nm).

In order to understand the influence of side chain on cellular interaction, the cellular uptake, intracellular localization and cytotoxicity were measured. The flow cytometry assay showed the cellular uptake of **4** was much higher than the other test compounds (Fig. 6(a)), which might be due to its positively side chain facilitating plasma membrane penetration. The comparatively poorer uptake of the other test compounds may thus be due to the negative side chain or the reduced solubility in the buffer solution. Since compound **4** shows a good ability to permeate cells, the intracellular localization of compound **4** was further investigated by confocal imaging (Fig. 6(b)), revealing its localization in the lysosomes.

The cytotoxicity of compounds **2**–**6** and CSTS to MCF-7 cells were tested. Interestingly, in the concentration range of 1.25–30 μM after incubation for 48 h (Figure S4) none showed appreciably cytotoxicity. This low cytotoxicity therefore highlights that these dicyanomethylene-substituted benzothiazole squaraines are potential molecular probe candidates for live cell studies.

## Discussion

Inspired by our previous report that CSTS has excellent selectivity to parallel G4s, and excellent optical properties for *in vivo* imaging, such as near-infrared excitation and emission wavelength, strong absorption and high fluorescence quantum yield, we developed a simple strategy for the synthesis of a library of analogues. In order to target squaraine derivatives that possess high selectivity and affinity to specific G4s, and that can be used for G4 *in vivo* studies, click chemistry methodology was employed to successfully functionalized the core structure of CSTS with two alkyne groups, and further linked to various azides to produce a range of dicyanomethylene-substituted benzothiazole squaraines. Our results showed that the click reaction did not affect the optical properties of the squaraine derivatives and their selectivity to parallel G4s. However, the side chains variation did significantly change the solubility, the affinity to different parallel G4s and the interaction with cells of squaraine derivatives. Of the compounds tested, compounds **4** and **5** which bear two tertiary amine groups and two phthalimide groups, respectively, exhibited the strongest binding ability to parallel G4s. Compound **4** also showed the highest cellular uptake, which we propose is facilitated by the tertiary amine groups. On the other hand, the negatively charged sulfonic acid groups or carboxyl groups of CSTS or compound **3**, respectively, may be the cause of the lower binding affinity to parallel G4s and lower cellular uptake of these compounds. Importantly, all of the compounds showed low cytotoxicity.

In summary, we have developed a click chemistry strategy to obtain different squaraine derivatives as probes for parallel G4 recognition. Overall, these results suggest that our strategy is effective for obtaining various squaraine derivatives that possess different binding affinities to different parallel G4s. The high efficiency of click chemistry makes this strategy suitable for high-throughput screening of appropriate ligands or probes for given G4s through combinatorial chemistry. The low cytotoxicity of dicyanomethylene-substituted benzothiazole squaraine derivatives makes it possible to seek a molecular probe for the detection a specific G4 in live cells or *in vivo*, as well as drug candidates target G4 in a specific oncogene.

## Methods

### DNA sequences

DNA sequences (Table 1) were purchased from Sangon Biotech Co., Ltd. (Beijing, China). RNA sequences were purchased from Rui Biotech Co., Ltd. (Beijing, China). The concentration of each DNA/RNA was determined by absorbance measurements at 260 nm, respectively on the basis of respective molar extinction coefficients using NanoDrop 2000 Spectrophotometer (Thermo Scientific, USA). Stock solutions of DNA were prepared in a buffer with 10 mM Tris-HCl, 100 mM KCl and 0.1 mM EDTA, pH 7.4. Stock solutions of RNA were prepared in a buffer with 20 mM Tris-HCl, 40 mM KCl and 0.1 mM EDTA, pH 7.4. For the 22AG in Na, a buffer with 10 mM Tris-HCl, 100 mM NaCl and 0.1 mM EDTA, pH 7.4, was used. Prior to measurements, the quadruplex samples were annealed at 95 °C for 5 min followed by slowly cooling to 4 °C and kept at this temperature overnight. Ssab DNA was prepared by heat denaturing and annealing the mixture of ssa and its complementary sequence (1:1). All DNA/RNA samples were stored at −20 °C when not being studied.

### Synthesis of compounds **1**–**6**

The synthesis route is illustrated in Fig. 1.

#### Synthesis of triethylammonium 3-(dicyanomethylene)-2-ethoxy-4-oxocyclobut-1-enolate (**1a**)

The compound was prepared as previously reported^33^. Malonodinitrile (0.66 g, 10 mmol) was added to a solution of 3,4-diethoxycyclobut-3-ene-1,2-dione (1.7 g, 10 mmol) in anhydrous benzene (45 mL) under stirring. Then, 1.3 mL Et_3_N was added dropwise. After stirring at room temperature for 30 min, the solvent was removed via rotary evaporation under reduced pressure and then purified by column chromatography (ethyl acetate:methanol = 10:1) to give the desired product (2.42 g, 83%). ^1^H NMR (400 MHz, MeOD) δ 4.73 (q, *J* = 7.1 Hz, 2 H), 3.24 (q, *J* = 7.3 Hz, 5 H), 1.46 (t, *J* = 7.1 Hz, 3H), 1.33 (t, *J* = 7.3 Hz, 8H).

#### Synthesis of 2-methyl-3-prop-2-ynyl-benzothiazol-3-ium bromide(**1b**)

2-methyl-1, 3-benzothiazole (5.07 g, 34 mmol) was added to a solution of propargyl bromide (11.9 g, 100 mmol) in CH_3_CN (30 mL). After stirring at reflux for 8 h, the solvent was removed via rotary evaporation under reduced pressure and then purified by column chromatography (dichloromethane:methanol = 10:1) to give the product (5.19 g, 57%) as a light green solid. ^1^H NMR (400 MHz, DMSO D6) δ 8.51(1H, d, J 8.0 Hz), 8.38 (1H, d, J 8.0 Hz), 7.95 (1H, t, J 8.0 Hz), 7.84 (1H, t, J 8.0 Hz), 5.78 (2H, s), 3.85 (1H, s), 3.28 (3H, s).

### Compound

#### (*Z*)-3-(dicyanomethylene)-2-((*Z*)-(3-(prop-2-yn-1-yl)benzo[d]thiazol-2(3H)-ylidene)methyl)-4-((3-(prop-2-yn-1-yl)benzo[d]thiazol-3-ium-2-yl)methylene)cyclobut-1-en-1-olate (**1**)

To a 250 mL, three-neck, round-bottom flash equipped with a Dean-Stark trap and a reflux condenser were added **1a** (1.46 g, 5 mmol), **1b** (3.2 g, 12 mmol), n-butanol (20 mL) and quinolone (20 mL) and toluene (100 mL). After stirring at reflux for 8 h, the solution was cooling to room temperature and added dropwise to excess amount of diethylether to form precipitation. The residue was collected by filtration and purified by column chromatography (dichloromethane:methanol = 70:1) to give the desired product (1.1 g, 42%) as a blue solid. ^1^H NMR (400 MHz, DMSO D6) δ 8.01(2H, d, J 8.0 Hz), 7.77 (2H, d, J 8.0 Hz), 7.56 (2H, t, J 8.0 Hz), 7.40 (2H, t, J 8.0 Hz), 6.28(2H, s), 5.14 (4H, s), 3.57(2H, s). HRMS (MALDI-TOF): calcd for C_29_H_16_N_4_OS_2_ [M]^+^ 500.0760, found:500.0763 m/z.

### General procedure for the synthesis of compouds **2–6**

To a solution of **1** (200 mg, 0.4 mmol) in DMSO were added freshly prepared solution containing sodium ascorbate (0.4 equiv), CuSO_4_·5H_2_O (0.2 equiv) and TBTA (0.2 equiv) in DMSO/H_2_O (10:1). Followed by the addition of azides **7–11** (3 equiv) was stirred at room temperature for 2 h and monitored by TLC. After the completion, the solution was added dropwise to excess amount of water to form precipitation. The residue was collected by filtration and purified by column chromatography using dichloromethane/methanol as eluent to give the desired product.

### Compound

#### (*Z*)-3-(dicyanomethylene)-2-((*Z*)-(3-((1-(4-hydroxybutyl)-1H-1,2,3-triazol-4-yl)methyl)benzo[d]thiazol-2(3H)-ylidene)methyl)-4-((3-((1-(4-hydroxybutyl)-1H-1,2,3-triazol-4-yl)methyl)benzo[d]thiazol-3-ium-2-yl)methylene)cyclobut-1-en-1-olate (**2**)

This compound was obtained as a blue solid (yield: 55%).^1^H NMR (400 MHz, DMSO D6) δ 8.25 (2H, s), 7.96(2H, d, J 8.0 Hz), 7.89 (2H, d, J 8.0 Hz), 7.55 (2H, t, J 8.0 Hz), 7.37 (2H, t, J 8.0 Hz), 6.26(2H, s), 5.53 (4H, s), 4.45 (2H, t, J 8.0 Hz), 4.36 (4H, t, J 8.0 Hz), 3.41 (4H, m), 1.83 (4H, m), 1.36 (4H, m. ^13^C NMR (151 MHz, DMSO D6) δ 173.44, 163.17, 161.44, 160.94, 141.28, 140.28, 128.18, 127.77, 125.35, 124.27, 123.23, 119.06, 114.23, 87.11, 60.41, 49.98, 41.91, 29.66, 26.88. HRMS (MALDI-TOF): calcd for C_37_H_34_N_10_O_3_S_2_ [M]^+^ 730.2251, found 730.2253 m/z.

### Compound

#### (*Z*)-2-((*Z*)-(3-((1-(3-carboxypropyl)-1H-1,2,3-triazol-4-yl)methyl)benzo[d]thiazol-2(3H)-ylidene)methyl)-4-((3-((1-(3-carboxypropyl)-1H-1,2,3-triazol-4-yl)methyl)benzo[d]thiazol-3-ium-2-yl)methylene)-3-(dicyanomethylene)cyclobut-1-en-1-olate (**3**)

This compound was obtained as a blue solid (yield: 58%). ^1^H NMR (400 MHz, DMSO D6) δ 8.26 (2H, s), 7.93(2H, d, J 8.0 Hz), 7.86 (2H, d, J 8.0 Hz), 7.53 (2H, t, J 8.0 Hz), 7.35 (2H, t, J 8.0 Hz), 6.24(2H, s), 5.52 (4H, s), 4.39 (4H, t, J 8.0 Hz), 2.22 (4H, m), 2.01 (4H, m). ^13^C NMR (151 MHz, DMSO D6) δ 173.47, 172.91, 162.68, 160.94, 160.42, 140.74, 139.89, 127.65, 127.26, 124.81, 123.86, 122.69, 118.54, 113.66, 86.62, 48.82, 41.39, 30.31, 25.06. HRMS (MALDI-TOF): calcd for C_37_H_30_N_10_O_5_S_2_ [M]^+^ 758.1837, found 758.1836 m/z.

### Compound

#### (*Z*)-3-(dicyanomethylene)-2-((*Z*)-(3-((1-(3-(dimethylamino)propyl)-1H-1,2,3-triazol-4-yl)methyl)benzo[d]thiazol-2(3H)-ylidene)methyl)-4-((3-((1-(3-(dimethylamino)propyl)-1H-1,2,3-triazol-4-yl)methyl)benzo[d]thiazol-3-ium-2-yl)methylene)cyclobut-1-en-1-olate (**4**)

This compound was obtained as a blue solid (yield: 49%). ^1^H NMR (400 MHz, DMSO D6) δ 8.25 (2H, s), 7.98(2H, d, J 8.0 Hz), 7.88 (2H, d, J 8.0 Hz), 7.55 (2H, t, J 8.0 Hz), 7.38 (2H, t, J 8.0 Hz), 6.25(2H, s), 5.54 (4H, s), 4.40 (4H, t, J 8.0 Hz), 3.05 (4H, m), 2.38 (12H, s), 2.06 (4H, m),. ^13^C NMR (151 MHz, DMSO D6) δ 173.51, 163.14, 161.44, 161.08, 141.26, 140.47, 129.23, 127.75, 125.47, 124.6, 123.32, 119.1, 114.2, 87.14, 63.12, 52.58, 45.97, 42.52, 29.46. HRMS (MALDI-TOF): calcd for C_39_H_40_N_12_OS_2_ [M]^+^ 756.2884, found 756.2884 m/z.

### Compound

#### (*Z*)-3-(dicyanomethylene)-2-((*Z*)-(3-((1-(4-(1,3-dioxoisoindolin-2-yl)butyl)-1H-1,2,3-triazol-4-yl)methyl)benzo[d]thiazol-2(3H)-ylidene)methyl)-4-((3-((1-(4-(1,3-dioxoisoindolin-2-yl)butyl)-1H-1,2,3-triazol-4-yl)methyl)benzo[d]thiazol-3-ium-2-yl)methylene)cyclobut-1-en-1-olate (**5**)

This compound was obtained as a blue solid (yield: 45%). ^1^H NMR (400 MHz, DMSO D6) δ 8.23 (2H, s), 7.96(2H, d, J 8.0 Hz), 7.88 (2H, d, J 8.0 Hz), 7.81 (4H, m), 7.77 (4H, m), 7.54 (2H, t, J 8.0 Hz), 7.37 (2H, t, J 8.0 Hz), 6.18 (2H, s), 5.51 (4H, s), 4.38 (4H, t, J 8.0 Hz), 3.55 (4H, t, J 8.0 Hz), 1.82 (4H, m), 1.53 (4H, m). ^13^C NMR (151 MHz, DMSO D6) δ 172.47, 168.36, 163.07, 161.37, 160.90, 141.29, 140.35, 134.73, 132.03, 128.19, 127.76, 125.35, 124.37, 123.39, 123.25, 118.96, 114.25, 87.04, 49.06, 40.89, 37.15, 27.41, 21.52. HRMS (MALDI-TOF): calcd for C_53_H_40_N_12_O_5_S_2_ [M]^+^ 988.2681, found 988.2682 m/z.

### Compound

#### (*Z*)-3-(dicyanomethylene)-2-((*Z*)-(3-((1-(2-(hydroxymethyl)-5-(5-methyl-2,4-dioxo-3,4-dihydropyrimidin-1(2H)-yl)tetrahydrofuran-3-yl)-1H-1,2,3-triazol-4-yl)methyl)benzo[d]thiazol-2(3H)-ylidene)methyl)-4-((3-((1-(2-(hydroxymethyl)-5-(5-methyl-2,4-dioxo-3,4-dihydropyrimidin-1(2H)-yl)tetrahydrofuran-3-yl)-1H-1,2,3-triazol-4-yl)methyl)benzo[d]thiazol-3-ium-2-yl)methylene)cyclobut-1-en-1-olate (**6**)

This compound was obtained as a blue solid (yield: 61%). ^1^H NMR (400 MHz, DMSO D6) δ 11.33 (2H, s), 8.41 (2H, s), 7.99(2H, d, J 8.0 Hz), 7.90 (2H, d, J 8.0 Hz), 7.81 (2H, s), 7.56 (2H, t, J 8.0 Hz), 7.38 (2H, t, J 8.0 Hz), 6.39 (2H, t, J 8.0 Hz), 6.25(2H, s), 5.58 (4H, s), 4.20 (2H, s), 3.67 (6H, m), 2.68 (6H, m), 1.80 (6H, s). ^13^C NMR (151 MHz, DMSO D6) δ 173.42, 169.07, 164.19, 163.14, 161.02, 150.87, 141.26, 140.65, 136.69, 128.25, 127.75, 125.40, 124.10, 123.30, 118.99, 114.19, 110.06, 87.20, 84.84, 84.32, 61.15, 59.90, 40.88, 37.54, 12.72. HRMS (MALDI-TOF): calcd for C_49_H_42_N_14_O_9_S_2_ [M+Na]^+^ 1057.2593, found 1057.2598 m/z.

### UV-visible spectroscopy

The aggregation of compounds in DMSO/Tris-HCl buffer mixtures was probed with the final concentration fixed at 4 μM, and the volume fraction of DMSO was varied from 0.0 (pure Tris-HCl buffer) to 1.0 (pure DMSO) with intervals of 0.2.

#### Absorbance spectra of compounds in the presence of different DNA

Small aliquots of stock solutions of compounds were added into the solution containing DNA in Tris-HCl buffer. The final concentration of compounds was 10 μM and DNA was 30 μM. After each addition of sample, the mixtures was allowed to be equilibrate for 1 h at room temperature, absorption spectra were collected.

### Fluorescence spectroscopy

Fluorescence Spectra were collected with excitation wavelength was set to *λ* = 680 nm and emission was recorded from 695–800 nm. A slit width of 5 nm for excitation and emission were employed. The aggregation of compounds in DMSO/Tris-HCl buffer mixtures was probed with final concentration fixed at 4 μM, and the volume fraction of DMSO was varied from 0.0 (pure Tris-HCl buffer) to 1.0 (pure DMSO) with intervals of 0.2.

#### Fluorescence spectra of compounds in the presence of different

*DNA:* small aliquots of stock solutions of compounds were added into the solution containing DNA in Tris-HCl buffer. The final concentration of compounds was 5 μM and DNA was 15 μM. For titration experiment, the final concentration of compounds was fixed at 4 μM in Tris-HCl buffer and the concentration of DNA was varied from 0 to 20 μM. After each addition of sample, the mixtures was allowed to be equilibrate for 1 h at room temperature, fluorescence spectra were collected. The apparent binding constants from the spectral titrations were derived using the following equation as reported previously^30^.

1 $$F/{F}_{0}=1+\frac{Q-1}{2}[M+1+x-\sqrt{{(M+1+x)}^{2}-4x}]$$

where, *F*_*0*_ and *F* correspond to the fluorescence intensity of free and DNA-bound compound at 710 nm, respectively. *M* = (*K*_a_ *C*_compound_)^−1^ and *x* = *nC*_DNA_(*C*_compound_^−1^), and *n* is the putative number of compound bound to a given DNA strand. The parameters *Q* and *M* were evaluated by Levenberg−Marquardt fitting routine in the Origin 8.5 software, whereas *n* was varied to obtain a better fit.

The absolute fluorescence quantum yieds (*Φ*) were measured using a FLS980 Spectrometer (Edinburgh instruments, UK) with an integrating sphere. After addition of 5-fold excess of ckit2 (5 μM) to compounds (1 μM), the mixtures were allowed to equilibrate for 1 h at room temperature. The emission spectra of the mixtures were recorded from 645–800 nm with excitation at 670 nm. The *Φ* values were derived using the equation *Φ* = (E − E_0_)/(S_0_ − S), E and E_0_ correspond to the integral of the emission region of sample and reference (Tris-HCl buffer only), S and S_0_ correspond to the integral of the excitation scatter region of sample and reference (Tris-HCl buffer only). The calculation of the integrals and the final calculation of *Φ* were use the F980 advanced software.

### Circular dichroism (CD) spectroscopy

Samples were taken with 1000 nm/min scan speed, 0.5 s response time in quartz cuvette of 10 mm optical path length. Each CD spectrum was the average of three scans at room temperature. Experiment carried out in Tris-HCl buffer without NaCl or KCl. 4 μM DNA was annealed in the presence of compounds (at [DNA]:[compound] ratios = 1:5) by heating at 95 °C for 5 min followed by cooling to 4 °C and kept at this temperature overnight before measured.

For melting studies, the DNA solutions (ckit2 dissolved in a buffer with 10 mM Tris-HCl, 5 mM KCl and 0.1 mM EDTA, pH 7.4, TBA dissolved in a buffer with 10 mM Tris-HCl, 5 mM NaCl and 0.1 mM EDTA, pH 7.4) were firstly prepared through heating at 95 °C for 5 min followed by cooling to 4 °C and kept at this temperature overnight. Then, 4 μM DNA (ckit2, TBA) were incubated with 5 molar equivalents of compounds (20 μM) for 1 h at room temperature. Following incubation, samples were melted from 25–95 °C taking measurements every 0.5 °C and the CD signal was monitored at 263 nm and 295 nm for ckit2 and TBA respectively at the heating rate of 1 °C/min.

### Flow cytometry assay

MCF-7 cells were seeded at a density of 5 × 10^5^ mL^−1^. After pre-incubation for 18 h in a 37 °C humidified incubator with 5% CO_2_, the medium was removed and fresh medium containing 5 μM compounds without FBS was added respectively. Then cells were incubated at 37 °C for 40 min. After washing with phosphate buffered saline (PBS pH 7.4) for three times, cells were incubated with EDTA for 15 min. Then culture medium was added and cells were centrifuged at 1000 rpm for 5 min, followed by resuspension in PBS for fluorescence analysis on the flow cytometry with FL4 channel. The data were analyzed with FlowJo VX.0.7 software.

### Confocal imaging

MCF-7 cells were grown on confocal dishes (35 mm × 12 mm, Φ20 mm glass bottom) for 24 h in a 37 °C humidified incubator with 5% CO_2_. After washing, cells were incubated with 1 mL of fresh medium containing compound **4**, Rhodamine 123 (mitochondria probe), LysoTracker Green DND-26 or DAPI at 37 °C for 30 min. Then the stained cells were washed with PBS three times and observed. Confocal images (512 × 512 pixels) were obtained using a 100 × objective lens and the images were overlaid using Olympus FV10-ASW 1.6 viewer software.

### Cytotoxicity assay

MCF-7 cells were seeded on a 96-well plate (5 × 10^3^ cells per well) and cultured for 18 h before treatment. Compouds were dissolved in DMSO and mixed with the culture medium. The maximum DMSO concentration in the cell culture medium was <0.5% v/v. Then compounds (1.25, 2.5, 5, 10, 20 and 30 μM) were added into cells, respectively, and incubated at 37 °C humidified incubator with 5% CO_2_ for an additional 48 h. Then the culture medium was removed and 100 μL of fresh medium (without FBS and penicillin/streptomycin) containing 10 μL of CCK-8 reagent (CCK-8, Dojindo, Japan) was added to each well. The absorbance at 450 nm was measured on a Spectra Max M5 after incubation at 37 °C /5% CO_2_ for 30 min. The cell viability rates (VR) were calculated according to the Equation (2):

2 $${\rm{V}}{\rm{R}}=\frac{{\rm{A}}-{{\rm{A}}}_{0}\,}{{\rm{A}}{\rm{s}}-{{\rm{A}}}_{0}}\times 100{\rm{ \% }}$$

where, A is the absorbance of the experimental group, A_s_ is the absorbance of the control group and A_0_ is the absorbance of the blank group.

## Electronic supplementary material

Development of squaraine based G-quadruplex ligands using click chemistry
