# Supplementary material for: Development of squaraine based G-quadruplex ligands using click chemistry
Source: Sci Rep. 2017 Jul 6;7:4766. doi: 10.1038/s41598-017-04344-x (PMC5500484; doi:10.1038/s41598-017-04344-x)
Supplement: Supplementary file 1 — Development of squaraine based G-quadruplex ligands using click chemistry [file 41598_2017_4344_MOESM1_ESM.pdf]

Development of squaraine based G-quadruplex ligands using click chemistry

Xin Zhang,[1,2] Yongbiao Wei,[1] Tao Bing,[1,2] Xiangjun Liu,[1,2] Nan Zhang,[1,2] Junyan Wang,[1,2] Junqing He,[1] Bing Jin,[1] and Dihua Shangguan[1,2,*]

[1] Beijing National Laboratory for Molecular Sciences, Key Laboratory of Analytical Chemistry for Living Biosystems, Institute of Chemistry, Research/Education Center for Excellence in Molecular Sciences, Institute of Chemistry, Chinese Academy of Sciences, Beijing, 100190, china

[2] University of the Chinese Academy of Sciences, Beijing 100049, China

* To whom correspondence should be addressed. Tel/Fax: 86-10-62528509; Email: sgdh@iccas.ac.cn

Materials and Reagents.

3,4 -diethoxycyclobut-3-ene-1,2-dione, malonodinitrile, and 2-methyl-1,3-benzothiazole were purchased from J&K Co., Ltd (Beijing, China). Azides were obtained from Amatek Scientific Co., Ltd (Suzhou, China). All other ordinary solvents and chemical reagents were used as received without further purification. Stock solutions of compounds (1mM) were prepared by dissolving them in DMSO and stored at -4 ℃. Further dilutions of samples to working concentrations were made with relevant buffer immediately prior to use.

Instruments.

^{1}H NMR spectra were recorded using TMS as the internal standard in DMSO-d_6 with a Bruker AV400 spectrometer. High resolution mass spectra (HRMS) were obtained on an autoflex III MALDI-TOF mass spectrometer (Bruker). UV-visible spectroscopic studies were carried out on a SpectraMax M5 instrument (Molecular Devices) using 10 mm path length quartz cuvette. The fluorescence spectroscopic studies were performed on a

Hitachi F-4600 fluorescence spectrofluorometer (Kyoto, JPN) using 10mm path length quartz cuvette. The CD spectra were measured over a wavelength range of 230-400 nm using a Jasco J-815 circular dichroism spectrometer (JASCO Ltd., JPN). Flow cytometry assay were carried out on a BD FACSCalibur flow cytometer. Cells were imaged under an OLYMPUS FV1000-IX81 confocal microscope (Olympus Corporation, Japan). For cytotoxicity assay, the absorbance at 450 nm was collected on Spectra Max M5.

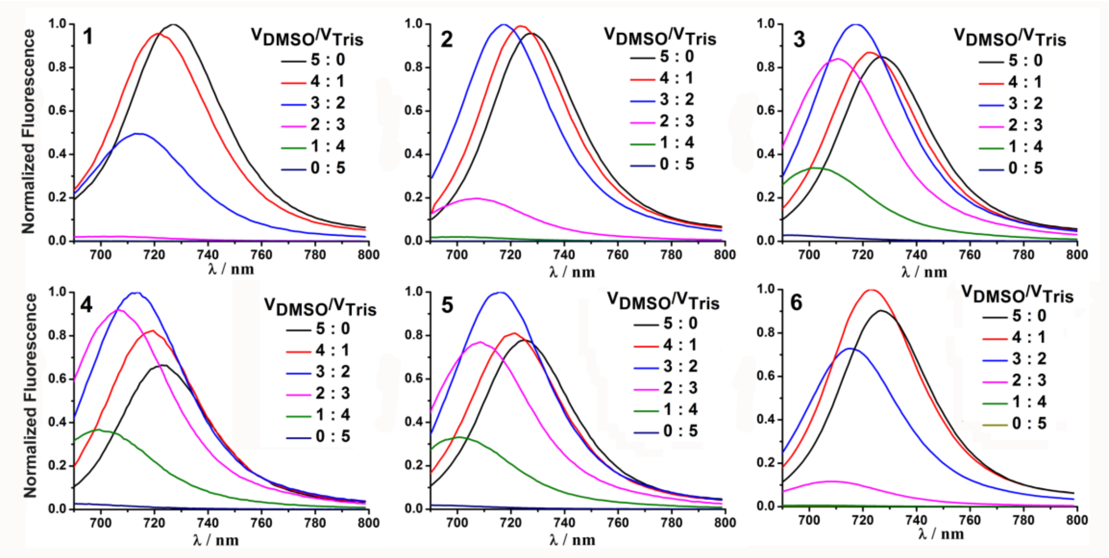

Figure S1. Fluorescence spectra of 4 μM compounds in mixed solvents of DMSO and Tris-HCl buffer.

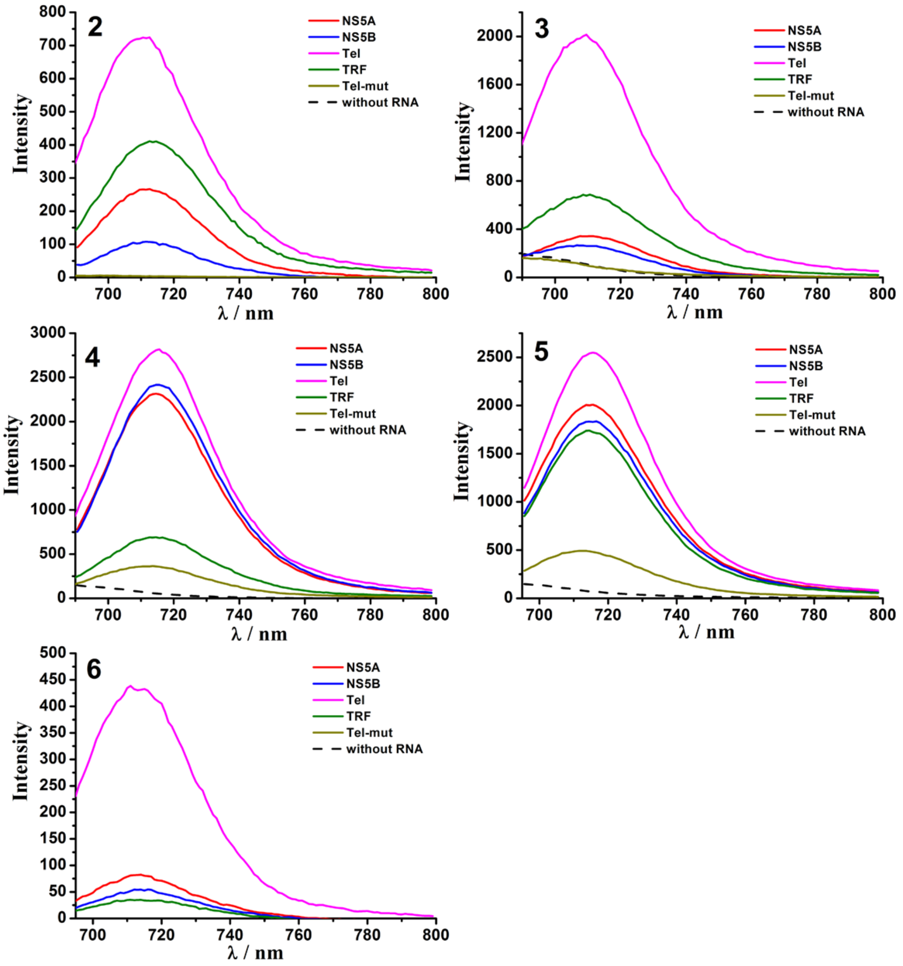

Figure S2. Fluoresence spectra of **2-6** (2 μM) with defferent RNA sequences (4 μM) in Tris-HCl buffer.

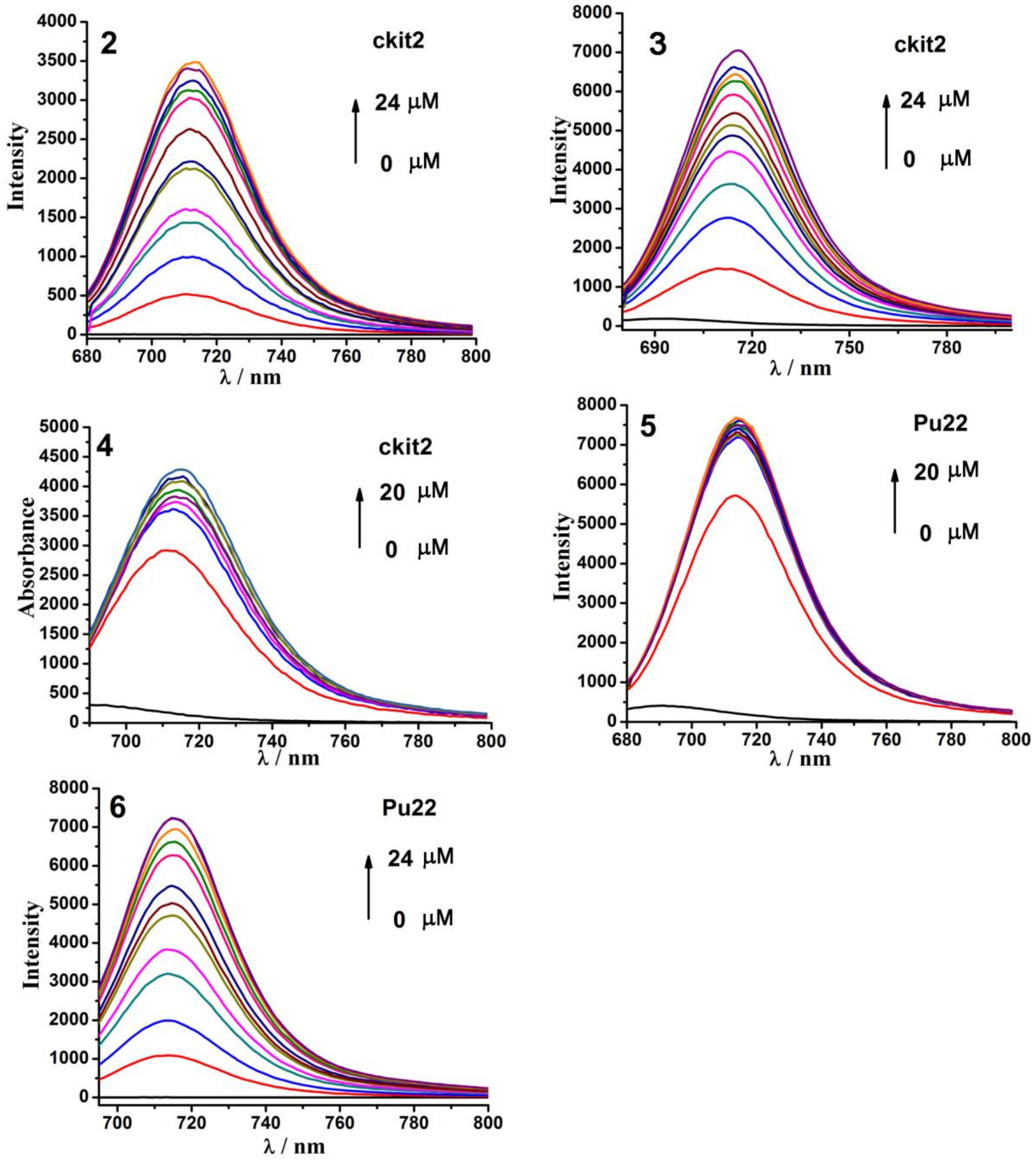

Figure S3. Fluorescence emission spectra of compound **2-6** (4 μM) with ckit2 (or Pu22) sequences in Tris-HCl buffer.

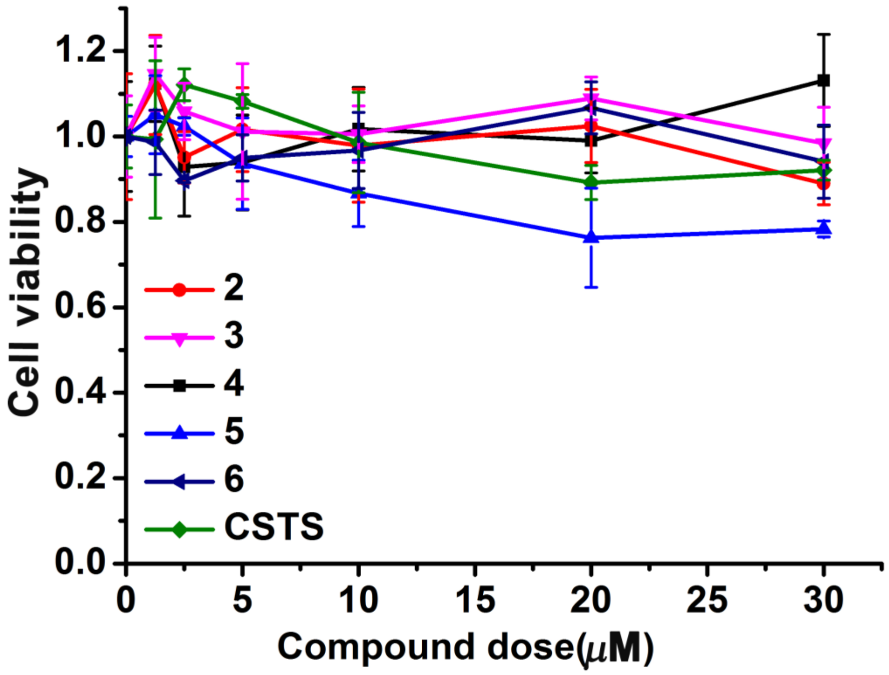

Figure S4. Cytotoxicities of compounds to MCF-7 cells. Data show the percentage of cell survival after incubated with compounds for 48 h.
